# A new species of the genus *Palpostilpnus* Aubert (Hymenoptera, Ichneumonidae, Cryptinae) from the Oriental part of China

**DOI:** 10.3897/zookeys.108.1123

**Published:** 2011-06-17

**Authors:** Mao-Ling Sheng, Gavin R. Broad

**Affiliations:** 1 General Station of Forest Pest Management, State Forestry Administration, Shenyang, Liaoning, 110034, China; 2 Department of Entomology, Natural History Museum, Cromwell Road, London SW7 5BD, UK

**Keywords:** *Palpostilpnus*, new species, Oriental Region, China, taxonomy, parasitoid

## Abstract

*Palpostilpnus brevis* Sheng & Broad, **sp.n.**, belonging to the tribe Phygadeuontini of the subfamily Cryptinae (Hymenoptera, Ichneumonidae), collected from Jiangxi Province, China, is described. A key to the described species of the genus *Palpostilpnus* Aubert, 1961, is provided.

## Introduction

Aubert (1961) established the genus *Townostilpnus* based on four species and one subspecies, which he separated into two subgenera, *Palpostilpnus* Aubert 1961, and *Townostilpnus* Aubert, 1961. Townes (1970) upgraded these subgenera to the generic level and placed them in separate subtribes. Other than the upgrading of one of Aubert's subspecies (*Townostilpnus (Palpostilpnus*) *striator papuator *Aubert, 1961) to species status (Gupta 1987), there have been no additions to the species level taxonomy of these genera. *Palpostilpnus* comprises three, and *Townostilpnus* two, described species, as originally included by Aubert (1961).

In this article, one new species belonging to *Palpostilpnus*, collected in Anfu and Quannan Counties, Jiangxi Province, China, is described. The holotype and two paratypes are deposited in the Insect Museum, General Station of Forest Pest Management (GSFPM), State Forestry Administration, People’s Republic of China. One paratype is deposited in the Natural History Museum, London, UK (BMNH).

Despite being placed in different subtribes by Townes (1970) (*Palpostilpnus *in Chiroticina, *Townostilpnus *in Gelina), the genera *Palpostilpnus *and *Townostilpnus *closely resemble each other. They were presumably separated by the form of the mandibles, which are subbasally swollen in *Townostilpnus*, with a basal, transverse groove. Contrastingly, the mandibles of *Palpostilpnus *are simple. *Townostilpnus *have a short ovipositor, but so does the species described here, and the length of the maxillary palp is variable in *Townostilpnus *but always very long in *Palpostilpnus*. It may be that these genera could be synonymized when the world fauna is better known. There are undescribed species of *Palpostilpnus *in the collections of the BMNH and other institutes but a thorough revision of the genus would require more collecting in various countries and much sorting of existing collections. We are describing this new Chinese species, the only *Palpostilpnus *so far found in China, so as to formally record the presence of the genus in China.

The morphological terminology is mostly that of Gauld (1991). Wing vein nomenclature is based on Ross (1936) and the terminology on (Mason (1986, 1990).

## *Palpostilpnus* Aubert, 1961 (New record for China)

### Palpostilpnus

Aubert, 1961

http://species-id.net/wiki/Palpostilpnus

Palpostilpnus Aubert, 1961. Bulletin de la Société Entomologique de Mulhouse, 1961:56. Type-species: *Townostilpnus* (*Palpostilpnus*)* palpator* Aubert. Designated by Townes, 1970.

#### Diagnosis.

Head and mesosoma short. Mandible small. Occipital carina reaching base of mandible. Maxillary palp reaching or almost reaching base of hind coxa. Sternaulus reaching mid coxa. Areas superomedia and petiolaris of propodeum combined. First tergum slender, without median dorsal carinae, spiracle near apical 0.22. Ovipositor very slender.

### Palpostilpnus brevis

Sheng & Broad sp. n.

urn:lsid:zoobank.org:act:207FA67A-9111-4A3C-8C6D-5E2E9AD342D3

http://species-id.net/wiki/Palpostilpnus_brevis

Figs 1–6 

#### Etymology.

The specific name is derived from the short ovipositor.

#### Material examined.

*Holotype*: female, CHINA: Quannan County, 700m, Jiangxi Province, 7 October 2008, leg. Mao-Ling Sheng (GSFPM). *Paratypes*: 1 female, same data as holotype (BMNH); 1 female, CHINA: Anfu County, 180m to 200m, Jiangxi Province, 12 October 2010, leg. Mao-Ling Sheng (GSFPM); 1 female, CHINA: Anfu County, 180m to 200m, Jiangxi Province, 1 November 2010, leg. Zhong-Ping Yu (GSFPM).

#### Diagnosis.

Clypeal suture very weak and indistinct. Postocellar line about as long as ocular-ocellar line. Hind wing vein 1/cu about 5 times as long as cu-a. Lateral carinae of area basalis are combined into one carina. Ovipositor sheath very short, approximately 0.5 mm. Second and third terga yellowish brown to reddish brown. Wings not banded, antennal flagellum with white band.

#### Description.

Female. Body length 4.0 to 4.5 mm. Fore wing length 3.5 to 3.8 mm. Antenna length 5.5 to 5.8 mm. Ovipositor sheath approximately 0.5mm.

##### Head.

Face (Fig. 2) approximately 1.9 times as wide as long, longitudinally convex centrally, forming narrow triangular area; with fine granulose texture and dense punctures, distance between punctures 0.2–1.0 times diameter of puncture; sublateral portion longitudinally concave. Clypeal suture very weak and indistinct. Clypeus evenly convex, almost smooth, with shallow and unclear punctures, 1.6 times as wide as long; apical margin evenly convex. Basal portion of mandible with weak and fine punctures; upper and lower margins almost parallel; teeth sharp, upper tooth approximately as long as lower tooth. Malar space with fine leathery granulose texture. Malar sulcus indistinct. Malar space approximately 0.67 times as long as basal width of mandible. Gena glossy, strongly convergent backwardly, with sparse, uneven and fine punctures. Vertex (Fig. 3) with fine leathery texture, posterior portion from behind ocelli to occipital carina slanted almost vertically, slightly concave. Postocellar line about as long as ocular-ocellar line. Frons with fine leathery texture, lower portion slightly concave. Antenna longer than body, with 34 flagellomeres, median portion slightly thickened, ratio of length from first to fifth flagellomeres: 1.5:1.2:1.0:0.9:0.8. Occipital carina complete.

##### Mesosoma.

Pronotum smooth, with sparse, fine punctures around margin; posterior portion with short transverse wrinkles. Collar very short. Epomia indistinct. Mesoscutum (Fig. 4) wide and short, comparatively convex, with fine leathery texture and indistinct punctures. Notaulus evident on front portion of mesoscutum. Scutoscutellar groove with short longitudinal wrinkles. Scutellum evenly convex, almost smooth, with very weak and indistinct leathery texture, anterior and lateral portion with fine granulose punctures. Postscutellum transverse, smooth. Mesopleuron (Fig. 5) mainly smooth, anterior portion with fine punctures; lower portion with leathery texture, punctures indistinct; posterior portion with fine transverse wrinkles and fine indistinct punctures. Epicnemial carina strong, almost straight, upper end reaching subalar prominence. Sternaulus distinct, nearly reaching hind margin of mesopleuron, far above lower posterior corner of mesopleuron. Metapleuron very long and narrow, with distinct punctures, distance between punctures 0.2–1.0 times diameter of puncture. Juxtacoxal carina distinct. Submetapleural carina complete. Wings brownish hyaline. Vein 1cu-a opposite 1/M, latter weakly bent forward. Vein 3rs-m absent. 2m-cu distal 2rs-m, about as long as distance between it and 2rs-m. 2m-cu inclivous, with two bullae. M+Cu comparatively arched. Vein 2-Cu slightly longer than 2cu-a. Hind wing vein 1/cu about 5 times as long as cu-a. Legs slender, comparatively long. Inner profile of basal portion of fore basitarsus distinctly bent. Hind coxa irregularly pyramidal, with fine and uneven punctures. Basal portion of hind tibia slender, gradually thick toward apex. Ratio of length of hind tarsomeres 3.3:2.6:2.2:1.0:0.8. Hind claws small. Propodeum (Fig. 6) steeply sloping from near anterior margin to posterior end; lateral longitudinal and pleural carinae distinct; areas superomedia and petiolaris combined, forming large, long area, costula located slightly before its middle; a longitudinal carina (fused lateral carinae of area basalis) between combined area and anterior margin of propodeum; posterior transverse carina absent; main median portion smooth, lateral area behind costula smooth, impunctate; lateral area before costula with sparse fine punctures and irregular short wrinkles; along carinae with irregular, indistinct, short wrinkles; spiracle small, oval, distance to anterior margin of propodeum 1.6 to 1.7 times its longest diameter.

##### Metasoma.

First tergum 3.6 to 3.8 times as long as apical width, smooth, petiole flat; postpetiole wide, anterior medially shallowly concave, lateral margins parallel; without median dorsal carina or dorsolateral carina; ventrolateral carina weak; spiracle small, round, slightly convex, at posterior 0.23. Second tergum smooth, widened posteriorly, about 0.6 times as long as apical width, 1.3 times as long as basal width; smooth. Third tergum 0.5 times as long as apical width, parallel-sided, finely punctate. Following terga indistinctly punctate. Ovipositor sheath short, approximately 0.18 times as long as hind tibia. Ovipositor very thin.

##### Color (Fig. 1).

Black, except the following. Dorsal profile of flagellomeres 6 to 11 white, ventral profile of apical flagellomeres yellowish brown. Scape, pedicel, base of first flagellomere, mandible except teeth, tegula, subalar ridge, legs, second and third terga yellowish brown to reddish brown. Maxillary and labial palpi, all coxae and trochanters yellowish white. Basal portion and outer profile of hind tibia, base of hind basitarsus blackish brown. Fourth and following terga, stigma and veins dark brown.

**Figures 1–6.**
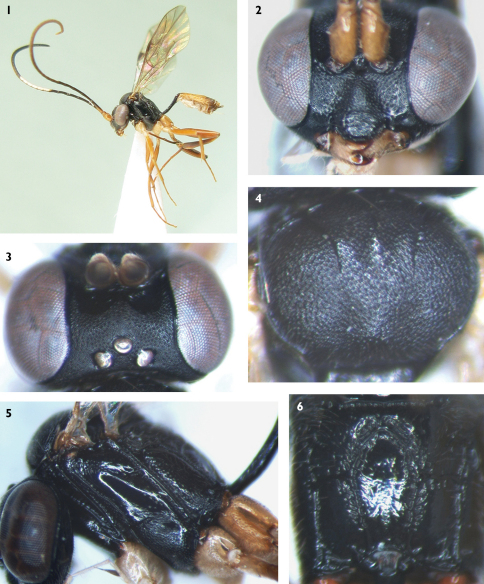
*Palpostilpnus brevis* Sheng & Broad, sp.n. **1** Body, lateral view **2** Face **3** Vertex **4** Mesoscutum **5** Mesopleuron **6** Propodeum.

#### Remarks.

Similar to *Palpostilpnus palpator* (Aubert, 1961) but can be distinguished from the latter by the following combination of characters: antenna with 34 flagellomeres; dorsal profile of flagellomeres 6 to 11 white; lower side of hind femur without basal tubercle; ovipositor sheath shorter than apical depth of metasoma. *Palpostilpnus palpator*: antenna with 22 flagellomeres; dorsal profile of flagellomeres without white; lower side of hind femur with a basal tubercle; ovipositor sheath much longer than apical depth of metasoma.

The new species can be inserted as follows in Aubert’s (1961) key to species, with the third couplet modified.

**Table:** 

3	Scutellum without wrinkles. Hind tibia without white, basal ring. Fore wing lacking brown, transverse band	3'
–	Scutellum with strong wrinkles. Hind tibia with white, basal ring. Fore wing with brown, transverse band	4
3'	Frons smooth. Antenna without white ring. Legs, including coxae, red.	3. *Palpostilpnus palpator* Aubert
–	Frons with fine leathery texture and unevenly punctuate. Dorsal median portion of antenna white. Fore and mid coxae white, hind tibia dark brown	*Palpostilpnus brevis* Sheng & Broad, sp.n.

## Supplementary Material

XML Treatment for Palpostilpnus

XML Treatment for Palpostilpnus brevis
